# CO Adsorption and Disproportionation on Smooth and Defect-Rich Ir(111)

**DOI:** 10.1021/acs.jpcc.2c01141

**Published:** 2022-04-08

**Authors:** Xia Li, Thomas Haunold, Stefan Werkovits, Laurence D. Marks, Peter Blaha, Günther Rupprechter

**Affiliations:** † Institute of Materials Chemistry, Technische Universität Wien, 1060 Vienna, Austria; ‡ Department of Materials Science and Engineering, Northwestern University, Evanston, Illinois 60208, United States

## Abstract

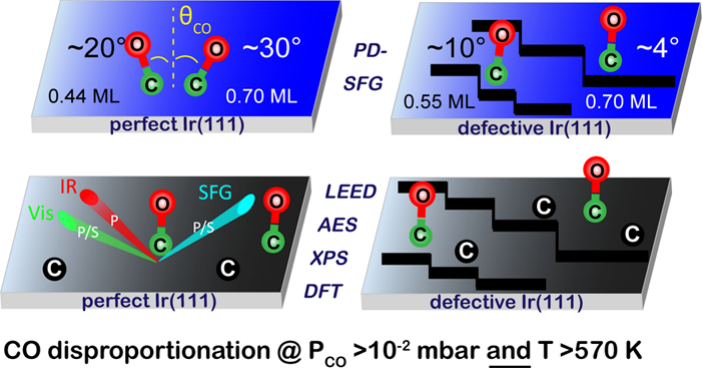

CO adsorption and dissociation on “perfect” and “defect-rich” Ir(111) surfaces were studied by a combination of surface-analytical techniques, including polarization-dependent (PPP and SSP) sum frequency generation (SFG) vibrational spectroscopy, low-energy electron diffraction (LEED), Auger electron spectroscopy, X-ray photoelectron spectroscopy (XPS), and density functional theory (DFT) calculations. CO was found to be ordered and tilted from the surface normal at high coverage on the “perfect” surface (e.g., θ = 30° at 0.70 ML), whereas it was less ordered and preferentially upright (θ = 4–10°) on the “defect-rich” surface for coverages of 0.55–0.70 ML. SFG, LEED, and XPS revealed that CO adsorption at low pressure/high temperature and high pressure/low temperature was reversible. In contrast, upon heating to ∼600 K in near mbar CO pressure, “perfect” and even more “defect-rich” Ir(111) surfaces were irreversibly modified by carbon deposits, which, according to DFT, result from CO disproportionation.

## 1 Introduction

Iridium surfaces have repeatedly attracted interest due to their thermo-catalytic properties.^1−4^ Iridium is also used in electro- and photocatalysis, employing Ir, Ir alloys (e.g., PtIr, RuIr, and PtNiIr), and IrO_2_ as nanoparticles or thin films.^5−10^ Previous surface science studies have shown that the Ir(100) surface may undergo a (1 × 1) → (5 × 1) surface reconstruction, which was lifted by the adsorption of small molecules (e.g., CO).^11−15^ Ir(110) also shows a (1 × 2) missing-row-type reconstruction with (111) micro-facets,^16^ while Ir(111) is reported to be the most stable surface.^17^

CO adsorption on Ir(111) has been studied for over 50 years by experimental surface science techniques such as low-energy electron diffraction (LEED), Auger electron spectroscopy (AES), temperature-programmed desorption (TPD), Fourier transform infrared reflection-adsorption spectroscopy (FT-IRAS), X-ray photoelectron spectroscopy (XPS), and sum frequency generation (SFG) spectroscopy,^1,4,18−26^ as well as by density functional theory (DFT).^24,27,28^ Two distinct LEED patterns indicated an ordered  structure at 1/3 ML^1,17−19^ and a (diffuse)  pattern at high coverage (7/12 ML^19^ and 2/3 ML^1,18^). With increasing CO coverage, FT-IRAS showed that the IR spectral intensities of linearly (on-top) bonded CO increased and vibrational frequencies blue-shifted,^20,24^ while in TPD, the desorption peaks shifted to lower temperature and different adsorption states formed, especially close to saturation.^18−20^ Such observations/trends are quite common for CO adsorption on metals, but we could recently demonstrate that upon increasing the coverage, CO was tilted on Ir(111) [at 0.77 ML by about 20° (DFT) or 36° (SFG)].^25^ Apparently, the CO adsorbate structures are strongly coverage-dependent, same as for Pd(111) surfaces (although CO populates hollow or bridge in addition to on-top sites on Pd).^29−32^

Herein, we reveal the effect of surface roughness on CO adsorption by extending the picture to “defect-rich” (sputtered) Ir(111) surfaces. Whereas conventional vibrational spectroscopy would detect only minor frequency shifts, polarization-dependent (PD-) SFG enables us to directly monitor the effect of defects on the molecular arrangement and orientation of the CO overlayer. SFG spectroscopy was carried out at a “near ambient pressure” of 1 mbar and at temperatures up to 600 K, which induced irreversible surface changes, likely originating from CO dissociation. Carbon deposits were detected by AES, and the reaction pathway via CO disproportionation was examined by DFT.

## 2 Methods

### 2.1 Basic Theory of Polarization-Dependent SFG

IR–visible SFG is a second-order surface-specific process as the effective second-order nonlinear susceptibility χ_eff_^(2)^ ≠ 0 at an anisotropic surface/interface. Polarization-dependent SFG spectra of adsorbed molecules are usually taken with PPP and SSP polarizations (the indices are defined in the order of SFG, visible and IR beams), which allows a quantitative analysis of molecular orientation.^25,33−39^ The SFG spectra can be fitted by Lorentzian lineshapes:
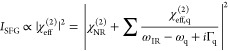
1where χ_NR_^(2)^ is the magnitude of the non-resonant susceptibility generated by the substrate. χ_q_, ω_q_, and Γ_q_ represent the resonance amplitude, frequency, and damping constant of the *q*th vibrational mode, respectively. When ω_IR_ is close or equal to ω_q_, the SFG intensity (i.e., *I*_SFG_) is enhanced and a vibrational peak appears in the SFG spectrum. The interfacial molecular orientations can be determined in an SFG experiment because the measured χ_eff_^(2)^ is related to the macroscopic second-order susceptibility in the laboratory coordinates (χ_ijk_^(2)^) by
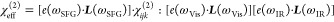
2where *e*(ω_*i*_) refers to the unit electric field vector and ***L***(ω_*i*_) is the Fresnel factor determined by the laser incidence and refraction angles, polarizations, and refractive indices.^33,34,36,40^ Furthermore, χ_*ijk*_^(2)^ is related to the microscopic hyperpolarizability tensor elements β_*i* ′ *j* ′ *k*′_^(2)^ in the molecular coordinate system through Euler transformation ⟨*R*_*ii*′_*R*_*jj*′_*R*_*kk*′_⟩ by^41^
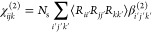
3Here, *N*_s_ is the effective molecular surface number density per unit. For CO with *C*_∞v_ symmetry, the molecules have a random azimuthal distribution, and the surface CO orientation (tilt angle θ, the CO molecular axis with respect to the surface normal) can be determined by measuring *I*_PPP_/*I*_SSP_ for a known molecular hyperpolarizability ratio *R* (i.e., *R* = β_aac_^(2)^/β_ccc_^(2)^ = β_bbc_^(2)^/β_ccc_^(2)^), assuming a δ-function for the orientation distribution.^25,42^

### 2.2 UHV Preparation/Analysis Chamber Coupled to a UHV-High-Pressure Cell for SFG Spectroscopy

All LEED/AES and SFG experiments were carried out in a custom-designed ultrahigh-vacuum (UHV) preparation/analysis chamber coupled to a UHV-to-atmospheric pressure-compatible SFG spectroscopic cell. The experimental setup has been described in detail previously.^43^

#### 2.2.1 UHV Preparation/Analysis Chamber Equipped with LEED/AES Optics

The UHV chamber is a stainless-steel vessel of about 40 L, which is pumped to a routine base pressure of 5 × 10^–10^ mbar by a turbomolecular drag pump Pfeiffer TMU, monitored by a hot-cathode gauge (Leybold IONIVAC ITR 90, GRAPHIX ONE controller). The chamber is further equipped with a four-grid retractable LEED/AES optics (SPECS ErLEED DN 150 CF) and a 3000D controller with a thoria (ThO_2_)-coated Ir filament that allows for a maximum of 10^–6^ mbar operation pressure. LEED patterns are recorded using a CMOS-sensor camera. The ErLEED 3000D power supply provides all necessary voltages to operate a LEED optics as a retarding field analyzer (RFA) for AES. For recording AES data, an integrated lock-in amplifier and RFC-PC software are used.

The Ir(111) single crystal was disk-shaped, with 8 mm diameter and 2 mm thickness. For a smooth/“perfect” surface, Ir(111) was pretreated by repeated cycles of sputtering with Ar^+^ ions (beam energy 1.2 keV at 5 × 10^–6^ mbar Ar, 30 min), oxidation (1 × 10^–7^ mbar O_2_, 30 min) at 800 K, and UHV annealing at 1050 K for 30 min, as described in refs (15, 18, 44, 45). This leads to ordered and clean surfaces, as confirmed by and described in detail in ref (15). LEED and AES were employed to verify the long-range order and cleanliness of the surface, respectively. For the sputtered “defect-rich” surface, freshly prepared “perfect” Ir(111) was sputtered with Ar^+^ ions using a beam energy of 1.2 keV at 5x10^–6^ mbar Ar for 40 min at 300 K (without subsequent annealing). For scanning tunneling microscopy (STM) images of a sputtered single crystal surface with three-dimensional islands exhibiting a very high density of steps and edges, one should refer to ref (46). CO of purity 4.7 (99.997%) from Messer Austria was used, further passed through a cold trap (AES confirmed that no Ni or Fe impurities were present^47^), with the CO overlayer structures characterized by LEED.

#### 2.2.2 UHV-High-Pressure Cell for SFG Spectroscopy

The freshly pretreated Ir(111) can be directly transferred from the UHV chamber to the SFG cell under UHV, avoiding contaminations. The SFG cell can be operated from 2.5 × 10^–8^ mbar to 1 bar pressure and at 100–800 K. SFG measurements were performed using a 20 ps mode-locked Nd:YAG laser system (EKSPLA, PL2241) with a fundamental radiation of 1064 nm (30 mJ/pulse, 50 Hz repetition rate). A tunable mid-infrared beam (with the photon energy ω_IR_) and a visible beam with a fixed wavelength of 532 nm were directed in a co-propagation geometry toward the Ir(111) surface (for details, see refs (39, 42, 43)), with incidence angles of 55° and 58.5° with respect to the surface normal, respectively. The pulse energy was 90–130 μJ for infrared between 1850 and 2150 cm^–1^ and 30 ± 5 μJ for visible. The SFG signal was collected/detected in the reflection direction with a photo-multiplier tube (PMT). The polarization of IR was kept as P and that of visible and SFG signal was switched between P and S using a Glan–Taylor prism and a half-wave plate. All spectra were normalized by the energy of visible and IR laser pulses and fitted using Lorentzian lineshapes (eq 1).

### 2.3 X-ray Photoelectron Spectroscopy

XPS experiments were carried out in another stainless-steel UHV chamber (35 L, base pressure < 5 × 10^–10^ mbar). A SPECS XR50 high-intensity nonmonochromatic Al/Mg dual-anode X-ray source and a Phoibos 100 hemispherical energy analyzer (EA) with a multichannel plate detector were used for XPS, as described in ref (48). Al K_α_ radiation (1486.61 eV) was used for the acquisition of XPS spectra. In this chamber, the sample preparation and surface order analysis by LEED followed the same procedure as described above.

### 2.4 Density Functional Theory

The disproportionation of two CO molecules on the Ir(111) surface into CO_2_ and C was studied by DFT using the augmented plane wave and local orbital (APW + lo) method as implemented in our WIEN2k code.^49,50^ The calculations of the present work used the generalized gradient approximation by Perdew, Burke, and Ernzerhof (PBE).^51,52^ We used a plane wave cutoff parameter *R*_MT_*K*_max_ = 5 for all calculations with a C atom and a properly scaled *R*_MT_*K*_max_ for the pure Ir surface, where *R*_MT_ refers to the smallest atomic sphere radius (2.2/1.05/0.95 bohr for Ir, O, and C atomic spheres, respectively). The final results were checked with *R*_MT_*K*_max_ = 6. A 4 × 4 × 1 (checked with 8 × 8 × 1) k-mesh was used, and the self-consistent field calculations and the atomic positions were fully relaxed until the forces were smaller than 1 mRy/bohr.

## 3 Results and Discussion

### 3.1 Surface Characterization of Clean Ir(111) by LEED, AES, and XPS

**Figure 1:**
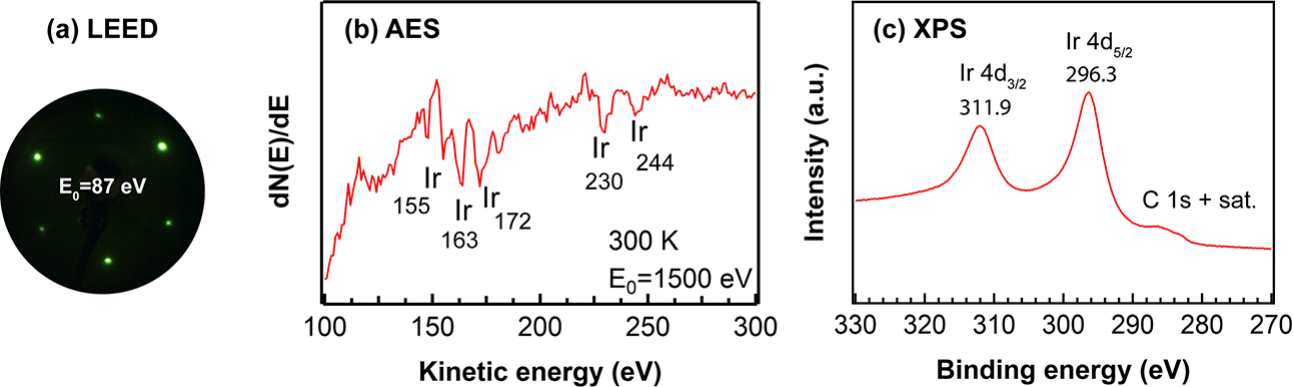
Characterization of clean Ir(111) at 300 K in UHV: (a) LEED pattern at *E*_0_ = 87 eV; (b) AES spectrum at *E*_0_ = 1500 eV; and (c) Ir 4d + C 1s region XPS spectrum.

Before CO adsorption, an ordered and clean Ir(111) was confirmed by LEED, AES, and XPS. As shown in Figure 1a, a hexagonal LEED pattern with sharp spots on a low background indicated a well-ordered Ir(111) surface. In AES spectra (Figure 1b), five Ir peaks in the absence of a carbon peak (expected around 272 eV^18,53^) demonstrated a clean surface. Unfortunately, the XPS C 1s analysis of C species on clean Ir(111) was somewhat hindered by overlapping satellite features of the Ir 4d peak due to the use of a nonmonochromatized X-ray source.^15^ As shown in Figure 1c, Al K_α3_ and K_α4_ satellites (at binding energies of 286.5 and 284.5 eV, respectively) of Ir 4d_5/2_ (at a binding energy of 296.3 eV) overlapped with the C 1s region. Still, significant amounts of carbon can be excluded.

### 3.2 LEED Patterns of CO Adsorption on Clean and Smooth Ir(111)

Previous LEED studies of CO on Ir(111) reported that  and  overlayer structures typically formed under UHV conditions.^19^ However, in the (>1.0) mbar regime, a more complex  structure forms instead.^44,54^ Our observations are consistent with these results.

**Figure 2:**
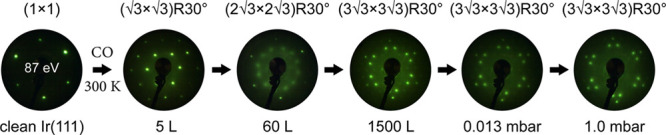
Evolution of LEED patterns at *E*_0_ = 87 eV of CO overlayer structures on Ir(111) as a function of CO exposure at 300 K: 5 and 60 L (1 Langmuir ≈ 10^–6^ mbar·s) were obtained by dosing CO at 5 × 10^–8^ mbar for 100 and 1200 s, respectively, and 1500 L was achieved by dosing CO at 5 × 10^–7^ mbar for 50 min. Pressures of 10^–2^ to 1 mbar were applied in the high-pressure SFG cell, with LEED taken after pump-down and transfer.

As shown in Figures 2 and S1, on the clean Ir(111) surface, a (1 × 1) LEED pattern formed, whereas upon CO chemisorption at 300 K and at 5 × 10^–8^ mbar pressure,  and  patterns were observed after 5 and 60 L exposure, respectively. When Ir(111) was exposed to 1500 L of CO (at 5 × 10^–7^ mbar), a  pattern was present.^44,54^ Further increasing the CO pressure to 1.0 mbar maintained the  overlayer structure. Accordingly, a CO- structure is formed not only at mbar pressure but also upon high exposure at relatively low pressure.

### 3.3 SFG Spectra of CO Adsorption on Ir(111): The “Perfect” vs “Defect-Rich” Surface

#### 3.3.1 Pressure-Dependent PPP and SSP Spectra

**Figure 3:**
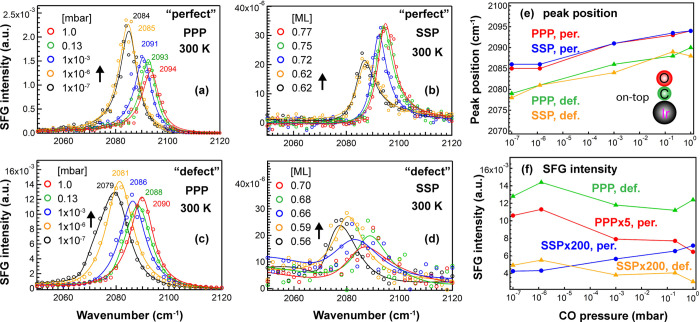
Pressure-dependent (10^–7^ to 1 mbar) SFG spectra of on-top CO on “perfect” and “defect-rich” Ir(111) at 300 K: (a,c) PPP and (b,d) SSP. All PPP and SSP peak positions and spectral intensities as a function of CO pressure are summarized in (e,f), respectively. Adapted in part with permission from ref (25). Copyright 2020 American Chemical Society.

Figure 3 compares the pressure-dependent SFG spectra of CO on the “perfect” and “defect-rich” Ir(111) surfaces at 300 K, in the range of 10^–7^ to 1 mbar, both for PPP and SSP polarization combinations. A previous combined IRAS/TPD study of CO/Ir(111) deduced a relationship between the IR peak position and the CO coverage,^20^ which is utilized herein to convert SFG peak positions to coverages for both surfaces as the differences in peak positions are small. Still, the coverages on the “defect-rich” surface may be slightly underestimated. In the 10^–6^ mbar range, the observed peak positions are well in line with IRAS studies of CO on Ir(111) and graphene-supported Ir clusters.^24^

A single peak, characteristic of on-top CO, was observed on both surfaces. As pressure increased from 10^–7^ to 1.0 mbar, the CO vibrational frequency moved from 2084 to 2094 cm^–1^ (coverage 0.62 to 0.77 ML) and from 2079 to 2090 cm^–1^ (coverage 0.56 to 0.70 ML) on “perfect” (Figure 3a,b) and “defect-rich” Ir(111) (Figure 3c,d), respectively. The frequency blue shifts can be attributed to the increasing dipole–dipole coupling and chemical shift.^20,55−57^ On the defective surface, the observed wavenumber is typically ∼5 cm^–1^ lower than on the smooth surface under the same conditions, reflecting the low-coordinated sites. Furthermore, in Figure 3c,d, assuming a second peak (<2080 cm^–1^), representing CO adsorbed on defects (e.g., steps, kinks, adatoms, and vacancies),^39,58−60^ was required to fit the experimental spectra (Figure S2). Accordingly, the full width at half-maximum (FWHM = 2Γ) of the on-top CO peak was 4 cm^–1^ larger (∼12 vs ∼8 cm^–1^) as the CO layer was less homogeneous on “defect-rich” surfaces.

For both surfaces, the CO peak positions acquired from PPP and SSP spectra were nearly identical (Figure 3e), but the spectral intensities (i.e., *I*_PPP_ and *I*_SSP_) exhibited different trends with increasing coverage/pressure: for CO on “perfect” Ir(111), *I*_PPP_ decreased, but *I*_SSP_ increased, whereas for CO on “defect-rich” Ir(111), both *I*_PPP_ and *I*_SSP_ hardly changed but overall decreased. Above 10^–3^ mbar, both vibrational frequency and intensity changed moderately because saturation was nearly reached. The fitting results of CO on “perfect” and “defect-rich” Ir(111) can be found in ref (25) and Table S1, respectively.

Our recent work about CO on smooth Ir(111) had pointed out that a decreasing *I*_PPP_ and an increasing *I*_SSP_ (Figure 3a,b,f**)** (smaller *I*_PPP_/*I*_SSP_) indicate an increasing tilt angle (θ), with θ increasing from 25° to 36° as the coverage increased from 0.62 to 0.77 ML.^25^ As CO tilted closer to the surface (i.e., larger θ), it yielded a relatively strong SSP signal (thus having a good signal-to-noise ratio) but caused a drop in the PPP signal. For CO on “defect” Ir(111), SSP shows a poor signal-to-noise ratio and is mostly due to the non-resonant background, whereas PPP was strong. Overall, this indicates a small θ so that CO is upright on defective Ir(111). *I*_PPP_ on the defective surface was stronger than on the perfect surface, likely due to a combined effect of the CO tilt angle, order, and coverage.

#### 3.3.2 Temperature-Dependent PPP and SSP Spectra

**Figure 4:**
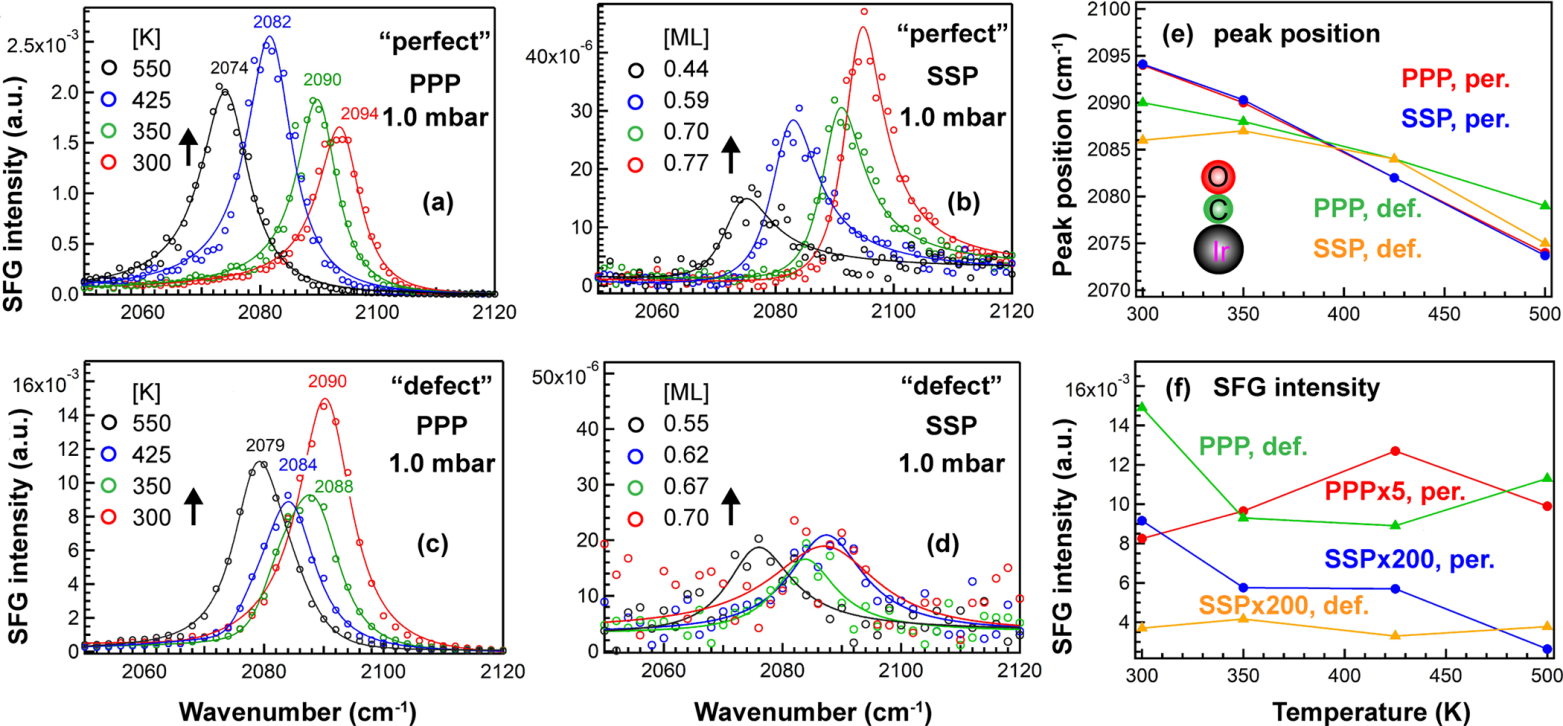
Temperature-dependent (300–500 K) SFG spectra of 1 mbar CO on “perfect” and “defect-rich” Ir(111): (a,c) PPP and (b,d) SSP. All PPP and SSP peak positions and spectral intensities as a function of substrate temperature are summarized in (e,f), respectively. Adapted in part with permission from ref (25). Copyright 2020 American Chemical Society.

SFG spectra were also acquired for both types of surfaces upon varying (lowering) the CO coverage by increasing the surface temperature from 300 to 500 K in a constant background of 1 mbar CO (Figure 4). As the temperature increased, the CO frequency red-shifted to low wavenumbers because of decreasing dipole coupling and different chemical shifts. Interestingly, the red shift on the defective surface was smaller than that on the smooth surface (11 vs 20 cm^–1^, respectively), which points to a smaller coverage change on the “defect-rich” surface that binds CO stronger. The fitting results of CO on the “perfect” and “defect-rich” Ir(111) can be found in ref (25) and Table S1, respectively.

As previously reported in detail for CO/“perfect” Ir(111), upon temperature increase, PPP and SSP changed oppositely (Figure 4a,b,f); that is, *I*_PPP_ first increased and then decreased, while *I*_SSP_ decreased gradually. The CO tilt angle decreased from 36° to 20° when the coverage decreased from 0.77 to 0.44 ML,^25^ so the increase of *I*_PPP_ was mainly attributed to a decreasing θ, and the decreasing *I*_SSP_ was due to the decreasing coverage and decreasing θ. In contrast, for CO on “defect-rich” Ir(111), PPP obviously decreased, whereas SSP changed only slightly. Similar to Figure 3, *I*_PPP_ was larger and *I*_SSP_ was smaller, once more confirming a small tilt angle θ on the defective surface.

#### 3.3.3 Quantitative Analysis of the CO Tilt Angle on Ir(111): “Perfect” vs “Defect-Rich”

**Figure 5:**
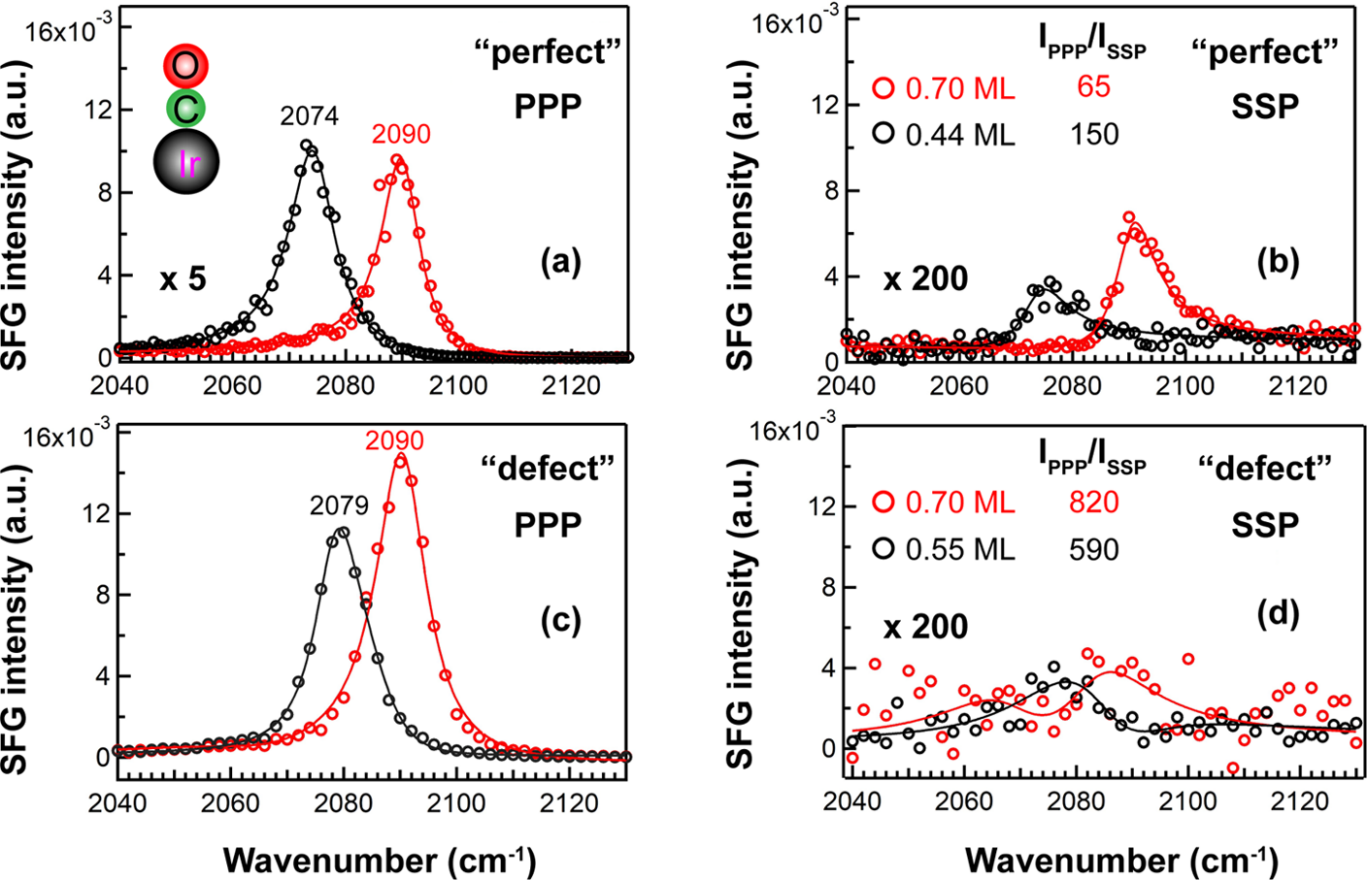
Selected SFG spectra of CO on “perfect” and “defect-rich” Ir(111): (a,c) PPP and (b,d) SSP.

In order to illustrate that the CO tilt angle on the “perfect” and “defect-rich” surfaces exhibited different coverage dependences, the PPP and SSP spectra were compared at two similar coverages, as shown in Figure 5. Notably, polarization-dependent SFG reveals a striking difference: for smooth Ir upon increasing the coverage (0.44 to 0.70 ML) (Figure 5a,b), *I*_PPP_ slightly decreased and *I*_SSP_ obviously increased. In contrast, for the defective Ir surface, *I*_PPP_ was distinctively larger and *I*_SSP_ was moderately larger at higher coverage (Figure 5c,d). This already illustrates a different trend.

**Figure 6:**
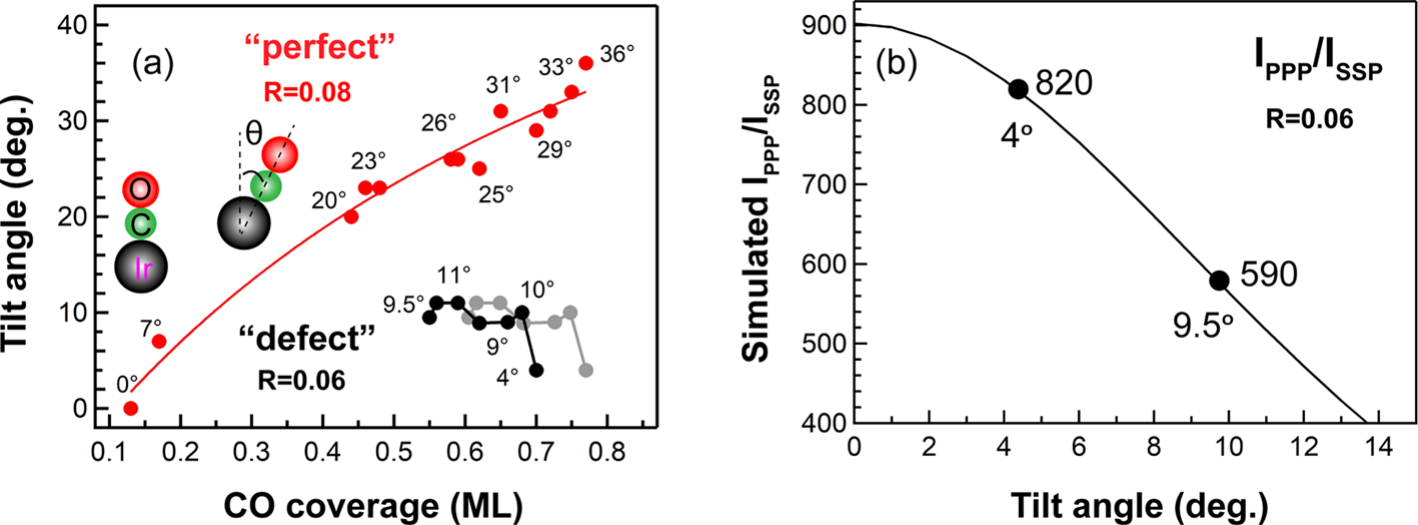
(a) Tilt angle (θ) of CO as a function of coverage, both for “perfect” and “defect-rich” Ir(111). The gray dots illustrate that the coverage on “defect-rich” may be slightly larger. (b) Simulated *I*_PPP_/*I*_SSP_ for CO/Ir(111) as a function of θ for a CO hyperpolarizability ratio *R* = 0.06. For parameters used in the simulations, one should refer to ref (25). Adapted in part with permission from ref (25). Copyright 2020 American Chemical Society.

As mentioned, the CO tilt angle can be deduced from *I*_PPP_/*I*_SSP_ if the *R*-value is known.^25,39^ Using CO on smooth Ir(111)^25^ at 0.13 ML (upright CO, θ = 0°) with *I*_PPP_/*I*_SSP_ = 520 as the reference, *R* was determined to be 0.08. Accordingly, for CO/“perfect” Ir(111),^25^*I*_PPP_/*I*_SSP_ of 150 and 65 at 0.44 and 0.70 ML coverage (Figure 5a,b) indicates corresponding tilt angles of 20° and 30°, respectively (Figure 6a**)**. Indeed, DFT calculations of the potential energy surface as a function of tilt angle for two CO molecules in nearest-neighbor positions in a 3 × 3 Ir(111) supercell had indicated that the energy increased dramatically when CO molecules came closer.^25^ Only when the CO molecules bent to the same direction (“concerted tilting”), the curve became flat.

For CO on “defect-rich” Ir(111), *I*_PPP_/*I*_SSP_ was 820 at 0.70 ML coverage (Figure 5c,d), and no reasonable θ can thus be obtained with *R* = 0.08. However, our recent work already reported the simulated *I*_PPP_/*I*_SSP_ versus θ for different *R* values (0.08, 0.07, and 0.06).^25^ With *R* decreased to 0.07 and 0.06, the maximum of *I*_PPP_/*I*_SSP_ increased to 670 and 900, respectively. Therefore, using *R* = 0.06 for 0.70 ML CO on the defective Ir(111), θ was determined to be 4° (Figure 6b). For 0.55 ML CO on defective Ir, *I*_PPP_/*I*_SSP_ was 590 pointing to a θ of 9.5°, that is, a somewhat higher tilt angle at lower coverage (opposite the trend on perfect Ir). Table S1 summarizes all tilt angles at different coverages for CO on “defect-rich” Ir(111) (based on the pressure- and temperature-dependent spectra in Figures 3 and 4). Except for θ = 4° at 0.70 ML, θ was ∼10° at all other coverages (0.67–0.55 ML, *R* = 0.06) (Figure 6a).

Summarizing the adsorption studies of CO on “defect-rich” Ir(111), the CO tilt angle was found to be small (4–10°) with only weak coverage depencence. The SFG spectral changes were mainly due to coverage changes. On the defective rough surface, the CO molecules seem to form a less-ordered overlayer and neighboring CO molecules are frequently located in different planes. This reduces the dipole–dipole repulsion so that “concerted tilting” is not induced and θ remains small. In summary, the surface roughness removed the strong coverage dependence of the CO tilt angle, observed on smooth Ir(111), so that on a rough surface CO was overall quite upright.

### 3.4 XPS, LEED, SFG, and AES Studies of CO Dissociation

DFT calculations have shown that CO cannot dissociate on Ir(111) due to a high effective barrier of 3.17 eV.^28^ This agrees with an ultraviolet photoelectron spectroscopy (UPS) study of Ir(111) in 10^–8^ Torr CO, which demonstrated that CO does not dissociate at an appreciable rate at 533 K.^61^ However, it was reported that heating Ir(111) to ≥650 K in ≥10^–6^ mbar CO^19^ or to ≥773 K in ≥10^–8^ mbar CO^18^ may result in significant dissociation. Therefore, in the following, a possible CO dissociation is examined for three different combinations of pressure and temperature by various surface-sensitive techniques: (i) XPS/LEED at low pressure, high temperature; (ii) SFG at high pressure, low temperature; and (iii) SFG/AES (post-reaction) at high pressure, high temperature.

#### 3.4.1 CO Dissociation at Low Pressure and High Temperature: XPS (10^–6^ mbar, 580 K) and LEED (10^–5^ mbar, 890 K)

**Figure 7:**
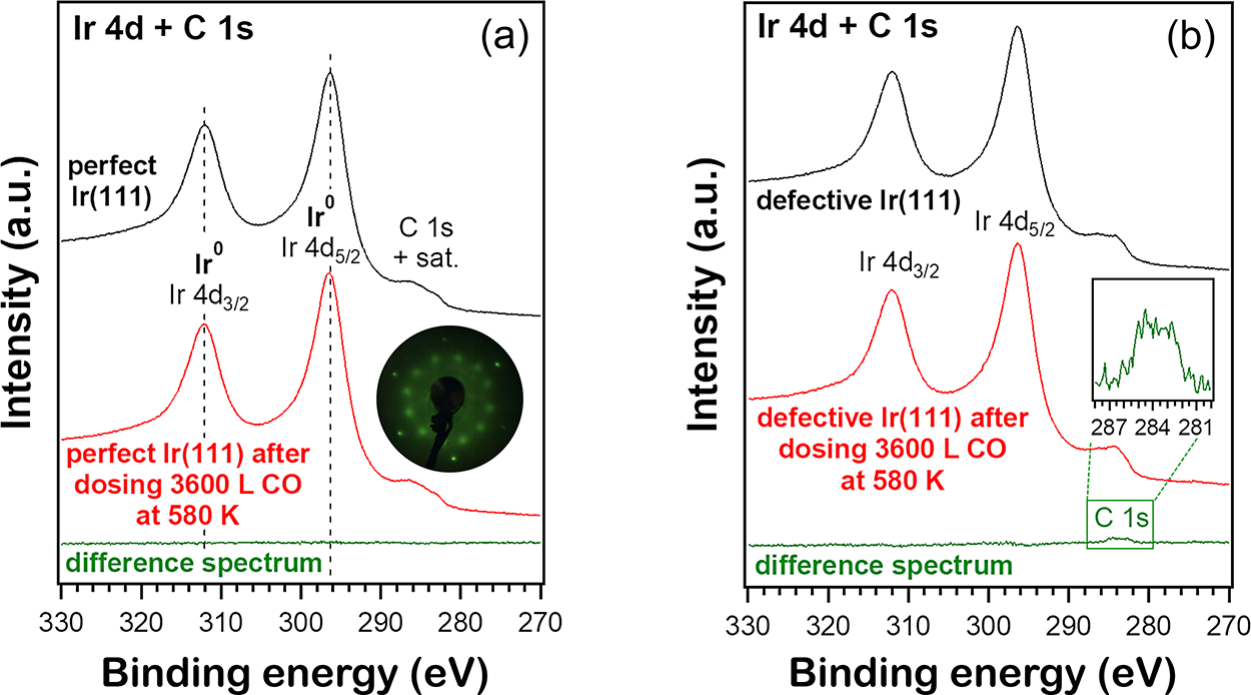
(a) Ir 4d + C 1s XPS spectra of perfect Ir (111) before and after annealing in 1 × 10^–6^ mbar CO at 580 K for 60 min, followed by cooling in UHV (spectra acquired at room temperature). The inset shows the subsequently acquired LEED pattern after dosing 300 L of CO at 300 K. (b) Corresponding Ir 4d + C 1s XPS spectra of defective Ir(111) before and after the same annealing as in (a).

First, Ir 4d + C1s XPS spectra of “perfect” Ir(111) were acquired before and after dosing 3600 L of CO (10^–6^ mbar CO for 3600 s) at 580 K (Figure 7a). The flat difference spectrum reveals that CO does not dissociate on Ir(111) under these conditions. After cooling to 300 K in UHV and dosing 300 L of CO, the -CO LEED pattern (Figure 7a, inset) verified the absence of CO dissociation. When the same experiment was repeated on defective Ir(111), only a very small amount of carbon was indicated by a tiny C 1s peak in the difference spectrum (Figure 7b).

**Figure 8:**
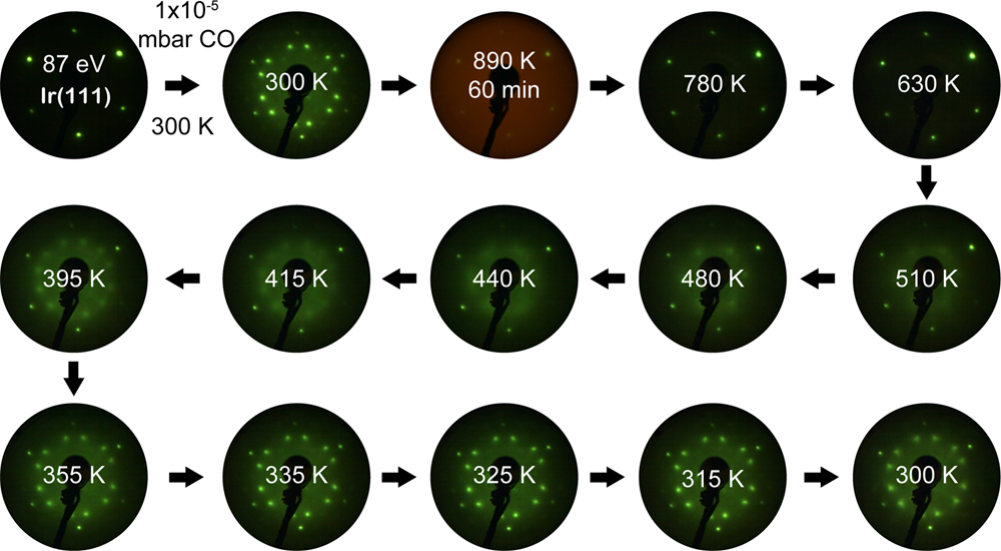
Evolution of LEED patterns at *E*_0_ = 87 eV of CO overlayer structures on “perfect” Ir(111) upon annealing in 1 × 10^–5^ mbar CO at 890 K for 60 min and stepwise cooling to 300 K.

Upon increasing the pressure to 10^–5^ mbar (for 60 min) and the temperature to 890 K (Figure 8), LEED showed complete CO desorption on perfect Ir(111) as the -CO pattern disappeared and (1 × 1)-Ir(111) appeared. The red background of the LEED pattern originated from glowing Ta-wires. Upon subsequent cooling to 630 K at 10^–5^ mbar, no CO chemisorption was observed. CO started to adsorb on the surface at 510 K forming a rather diffuse  structure until relatively sharp spots appeared at 480 K. At 415 K, a  structure began to develop. When the temperature was decreased from 355 to 300 K,  occurred, reestablishing the pattern at 300 K before heating. Therefore, the reversible LEED patterns before and after heating to 890 K in 10^–5^ mbar CO also indicate that CO does not dissociate upon the combination of low pressure and high temperature. No meaningful LEED patterns can be observed on the sputtered surface, preventing a comparison.

#### 3.4.2 SFG Study of CO Dissociation at High Pressure (1.0 mbar) and (Relatively) Low Temperature (500 K)

**Figure 9:**
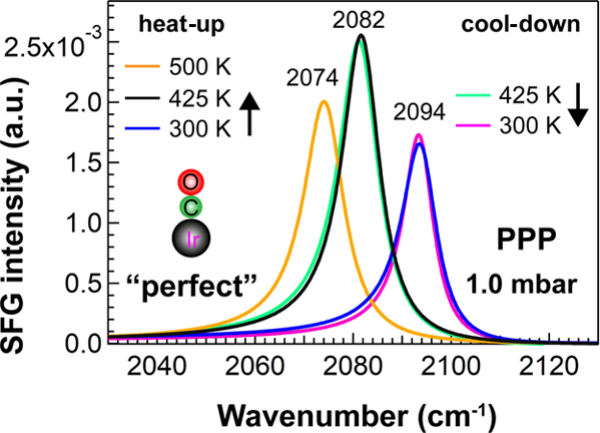
Selected PPP spectra of CO on “perfect” Ir(111) upon heating to 500 K and cooling in 1.0 mbar CO. For clarity, only the fitted lines are shown.

When the Ir surfaces were stepwise heated in 1 mbar CO from 300 to 500 K, the SFG spectra acquired upon heating and cooling were identical; that is, the spectra were fully reversible (Figure 9). This suggests that the surface does not change; that is, CO dissociation was absent.

#### 3.4.3 SFG Study of CO Dissociation at High Pressure (≥10^–2^ mbar) and (Relatively) High Temperature (≥575 K)

**Figure 10:**
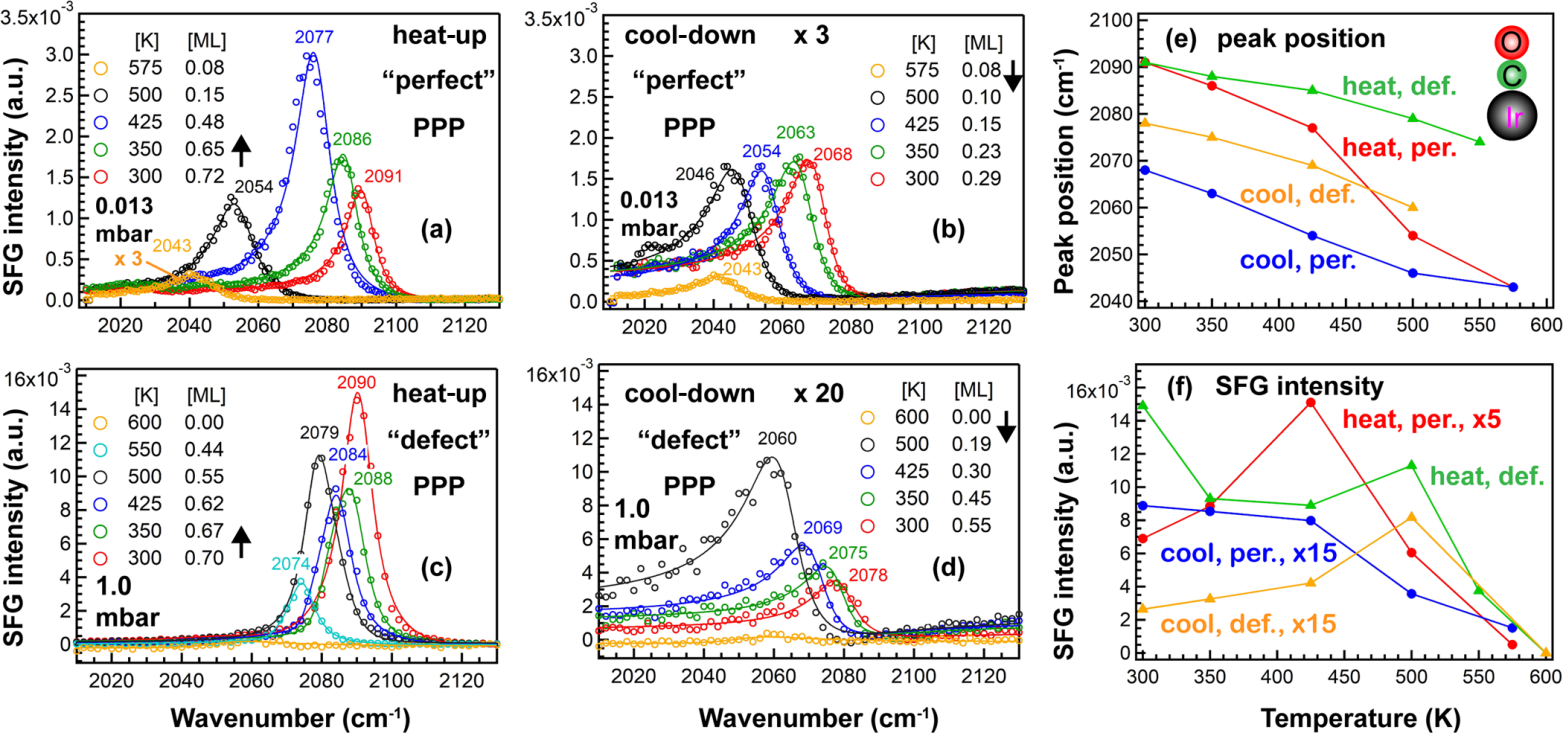
Temperature-dependent (300–600 K) SFG (PPP) spectra of CO on “perfect” (0.013 mbar) and “defect-rich” (1.0 mbar) Ir(111) upon stepwise heating (a,c) and subsequent stepwise cooling to 300 K (b,d). All PPP and SSP peak positions and spectral intensities of on-top CO as a function of temperature acquired upon heating and cooling are summarized in (e,f), respectively.

Interestingly, when the substrate temperature reached 575 K, the SFG spectra acquired during heat-up were still “as-expected” (Figure 10a,b), whereas the spectra taken during cool-down were very different from the previous ones under the same nominal conditions; that is, there were irreversible changes in the spectra.

Figure 10 again compares smooth and “defect-rich” Ir(111) upon heat-up and cool-down. When comparing SFG PPP spectra of CO on “perfect” Ir(111) before and after reaching 575 K, it is apparent that (Figure 10a,b,e,f) (i) the vibrational frequencies were lowered by about 23 cm^–1^, (ii) the spectral intensities were at least three times smaller (Table S2), and (iii) the spectral lineshapes became (more) asymmetric. All features indicate a strong modification of the Ir(111) surface, once reaching 575 K in 1.3 × 10^–2^ mbar CO. Strong restructuring of Ir(111) and/or CO dissociation may be responsible for the observed effect. Similar results were also found in an SFG study of CO adsorption and dissociation on Pt(111).^62,62^ When Pt(111) was heated in 400 Torr CO to 823 K and then cooled to room temperature, an apparent hysteresis in CO vibrational frequency and a decrease in intensity were observed due to carbon formation at high temperature. The AES spectra of the Pt(111) surface after exposure to 400 Torr of CO at 673 K showed a notable carbon peak, also indicating CO dissociation on Pt(111) at this temperature.^62,63^ For Pt thin films and nanoparticles in 10 mbar CO, dissociation was observed by SFG and near ambient pressure (NAP-)XPS upon heating to 550 K.^64^ On smooth and sputtered Pd(111), no indications of CO dissociation were observed even after hours in 0.1 mbar CO, likely due to the temperature limit of 400 K.^65−67^ It is well documented that carbon perturbs the electronic and geometric structure of stepped surfaces, which leads to poisoning.^68,69^

Upon cool-down (Figure 10b), the peak positions indicated a coverage increase from 0.1 to 0.29 ML, but the spectral intensities remained nearly unchanged. Based on our former work showing that CO was tilted at high coverage,^25^ the expected increase in PPP intensity due to increasing coverage seems compensated by the loss of intensity due to an increasing tilt angle. Unfortunately, the SSP signals were too weak for detection after heating because even before heating *I*_SSP_ was >35 times smaller than *I*_PPP_(25) (and after heating even *I*_PPP_ decreased three times).

The spectral reversibility was also investigated for the “defect-rich” Ir(111) surface. Figure 10c,d shows the PPP spectra before and after heating to 600 K at 1.0 mbar CO. Analogous to the smooth surface, after reaching 600 K, the spectra showed a red shift of about 13 cm^–1^ and the lineshapes changed to asymmetric. Note that the intensities were reduced even ∼20 times. Apparently, although similar in tendency, the changes were much stronger for the defect-rich surface, which may result from the roughness and/or the higher CO pressure (this will be further discussed below).

For CO/Ir(111), previous AES studies reported significant CO dissociation at >773 K at 10^–8^ mbar^18^ or at ≥650 K at 1.33 × 10^–6^ mbar.^19^ Accordingly, at 10^–2^ to 1.0 mbar CO used herein, dissociation may occur at comparably lower temperature. CO may also dissociate on Ir adatoms or clusters mobilized by CO at high pressure, that is, CO-induced surface roughening, as observed for CO on Cu(100)^70^ or Pt nanoparticles^71^ at mbar CO pressure even around room temperature.

CO dissociation or disproportionation may thus be responsible for the irreversible changes that only occurred when heated higher than 570 K in (near) mbar CO pressure. The activation barriers of direct CO dissociation on noble metals were computed by DFT to be rather high,^28^ but the barriers of CO disproportionation via the Boudouard reaction 2CO ↔ CO_2_ + C on low-coordinated sites have been shown to be much lower (e.g., on Rh,^72^ Cu,^73,74^ or Pt^64^).

#### 3.4.4 SFG, LEED, and AES Studies of Carbon Deposits

**Figure 11:**
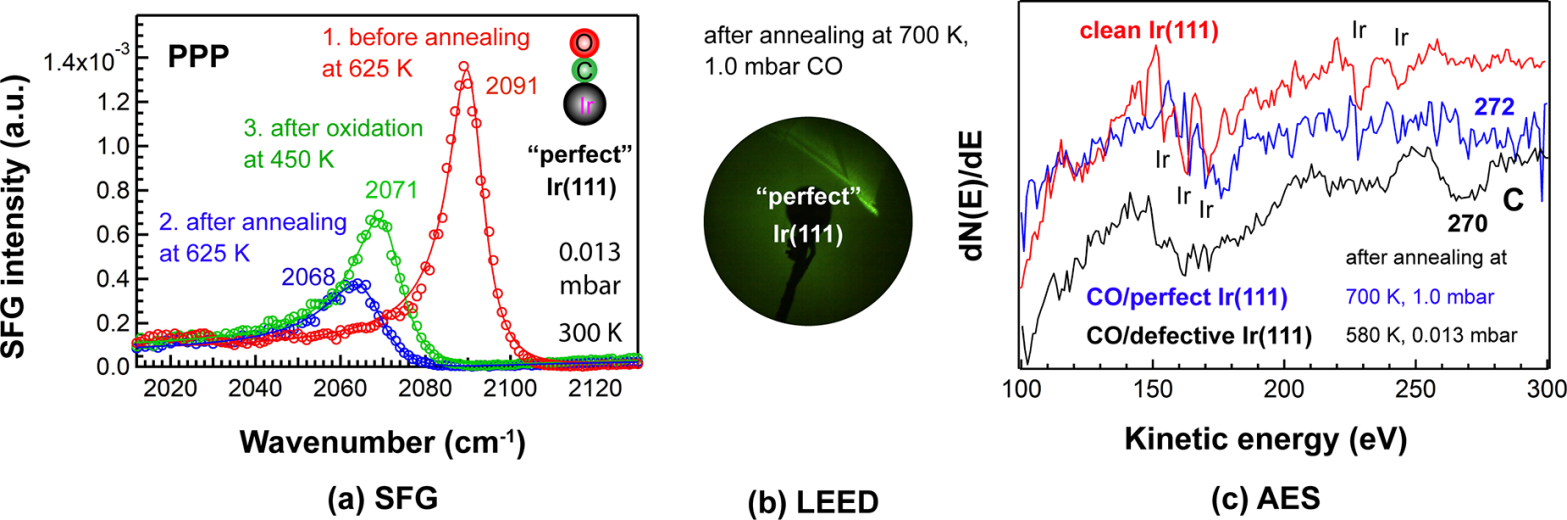
(a) Room-temperature PPP-SFG spectra of CO adsorption on “perfect” Ir(111) at 0.013 mbar, before (red) and after (blue) annealing at 625 K in CO, and after oxidation (green; 5 × 10^–3^ mbar O_2_ at 450 K). (b) LEED pattern at *E*_0_ = 87 eV acquired after exposing clean Ir(111) to 1.0 mbar CO at 300 K, heating in CO to 700 K for 60 min, and cooling to room temperature in UHV; (c) AES spectra at *E*_0_ = 1500 eV.

As seen in Figure 11a, after heating perfect Ir(111) to 625 K in 0.013 mbar CO, the room-temperature PPP spectrum of on-top CO originally centered at 2091 cm^–1^ (red) was red-shifted to 2068 cm^–1^ (blue), accompanied by a 3.5-fold intensity loss. To confirm that carbon was formed on the Ir(111) surface at 625 K, an oxidation experiment was then performed. As expected, after oxidation at 450 K in 5 × 10^–3^ mbar O_2_, not only the peak position moved to higher wavenumbers (2071 vs 2068 cm^–1^), but also the spectral intensity increased (green vs blue) because carbon was (partially) removed by oxidation. This “partly reversible” spectrum suggests that CO dissociation did occur at 625 K in 0.013 mbar of CO. Analogous to defect-rich Ir(111) (Figure 10c,d), a dissociation experiment was also carried out in 1.0 mbar CO (Figure 11b,c).

Carbon deposits increase the LEED background intensity and can be detected by AES,^19^ while the dissociated oxygen is removed from the surface via reaction with gaseous CO. Accordingly, a LEED pattern was measured after dosing 1.0 mbar CO at 300 K and annealing at 700 K for 60 min in the high-pressure cell. As seen in Figure 11b, after cooling in UHV, the LEED pattern was fuzzy, indicating that the surface was covered by carbon. Subsequently, an AES spectrum was obtained (Figure 11c). Compared to the AES spectrum of clean Ir(111) (Figure 1b), apart from the Ir-peaks becoming weak, a broad carbon peak appeared centered at around 272 eV. The carbon peak was even stronger when the experiment was repeated in 0.013 mbar CO on “defective” Ir(111) (270 eV), suggesting that surface roughness is more crucial than CO pressure. Thus, CO dissociates on Ir(111) but clearly requires high pressure and high temperature. Carbon quantification by XPS, either in situ^64−66,75^ or ex situ,^70^ before/after high-pressure CO exposure is planned for the future.

### 3.5 DFT Study of CO Disproportionation

**Figure 12:**
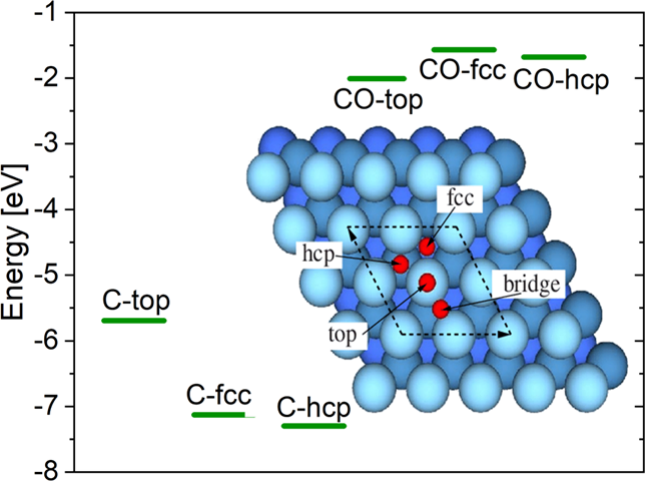
Adsorption energies of C and CO on Ir(111) at 0.25 ML coverage on various high-symmetry sites, which are indicated in the inset.

CO dissociation on Ir(111), still being rather unexpected, was further investigated computationally. DFT was used to study the disproportionation on Ir(111): 2CO ↔ CO_2_ + C.^49,50^ The C and CO adsorption as well as the disproportionation of CO was modeled by 2 × 2 supercells of Ir(111) (using the theoretical equilibrium lattice parameter of 3.874 Å) with five layers of Ir (Figure 12). In good agreement with previous calculations,^24,27,28^ a preference for CO adsorption on top sites and on hcp sites for C atoms was found (Table 1). The C adsorption is very strong, and C can be removed from the surface only under harsh oxygen-rich conditions. At larger coverages, however, also the hollow fcc or hcp sites can be occupied by CO, and overall, the binding energy of CO on Ir(111) gets reduced.^25^

**Table 1:** Adsorption Energies (eV) of C and CO on Ir(111) at Various Sites and 0.25 ML Coverage^a^

site	top	fcc	hcp
C	–5.69	–7.13	**–7.30**
CO	**–2.01**	–1.57	–1.68

a The most stable sites are marked in bold.

**Figure 13:**
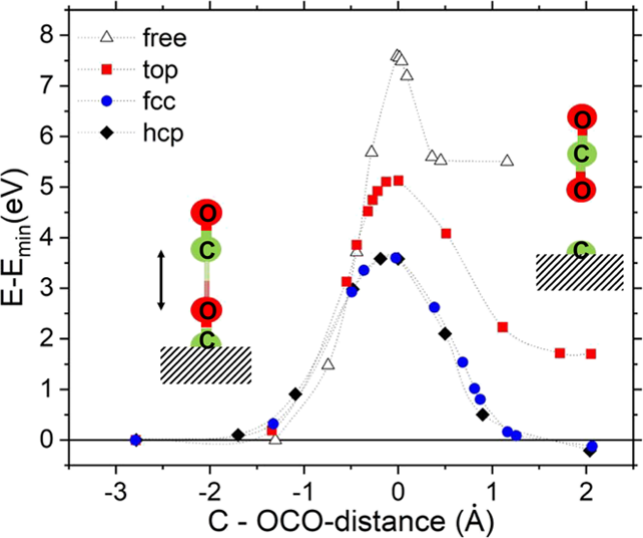
Energy barrier (in eV) for the Boudouard reaction 2CO ↔ C + CO_2_ in free space or with CO adsorbed on Ir(111) at three different sites. The energy zero is set to a large CO–CO distance (left side); the C–OCO distance (indicated by an arrow) is set to zero at the transition barrier. The lines are just guides to the eye.

In order to determine the dissociation barrier for CO, we put a second CO molecule on top but far away from the adsorbed CO (energy zero in Figure 13). Then, we used a constraint minimization technique, where the *x* and *y* coordinates of CO are fixed, but *z* of all atoms can relax. In addition, the C–OCO distance (see Figure 13) is automatically slowly reduced by an increasing pseudo force until the energy reaches a maximum and the pseudo forces change sign, which indicates the transition state. During this approach, the adsorbed CO molecule on the fcc or hcp site gets pushed deeper into the surface, increasing the Ir–C interaction and weakening the C–O bond. On the contrary, for the CO-top position, the Ir atom beneath CO acts like a hard wall and much less relaxation is possible. This leads to a relatively small activation energy of about 3.6 eV for the hcp and fcc sites (Figure 13), whereas more than 5.1 eV is necessary for the top position. However, this is still a drastic reduction compared to a reaction in free space with a barrier height of 7.6 eV. From Figure 13, it is also evident that the end products (C + CO_2_) have unfavorable energies for the reaction in free space and on the Ir-top position (since C on top of Ir has the lowest C adsorption energy), whereas they are slightly favored for hcp/fcc sites since C is very strongly adsorbed there.

These computational results help to verify the experimental findings. At low coverage, CO adsorbs only at the top site and the activation energy is so high that CO would desorb from the surface before a disproportionation is possible. However, at high CO partial pressure and high CO surface coverage, the hcp/fcc sites are partially occupied, and with increasing temperature, the adsorbed CO molecule on these sites can react with gas-phase CO forming CO_2_ and leaving a C atom behind, which remains strongly adsorbed and poisons the surface. CO adsorbed on hcp/fcc sites was experimentally not observed,^25^ suggesting that it is a transient species in the process.

## 4 Conclusions

We have used surface-sensitive PD-SFG, LEED/AES, XPS, and DFT calculations to study CO adsorption and dissociation/disproportionation on both smooth and defect-rich Ir(111) surfaces. PD-SFG showed that, in contrast to the strong coverage dependence of the CO tilt angle on smooth Ir(111) (i.e., CO tilted 30° at 0.70 ML), on the defect-rich surface, CO preferred standing upright at high coverage (4° at 0.70 ML). When the coverage ranged from 0.67 to 0.55 ML on defective surfaces, the CO tilt angle remained constant at ∼10° but still rather small. CO forms three different overlayer structures as observed by LEED:  ×  and  ×  at low CO exposure and  ×  at high CO exposure/pressure. XPS and LEED studies indicated that there is no CO dissociation at low pressure (10^–6^ mbar)/high temperature (890 K) or high pressure (1.0 mbar)/low temperature (500 K). However, upon heat-up (300 to ∼600 K) and cool-down (∼600 to 300 K) in a background of ∼1 mbar CO, the obtained irreversible SFG spectra implied that CO dissociated on smooth and especially defective Ir(111), yielding carbon deposits. SFG spectra upon carbon oxidation and AES spectra of Ir(111) after annealing in 1.0 mbar of CO at 700 K indirectly and directly confirmed the formation of surface carbon species, respectively. DFT calculations suggested that at high pressure, CO adsorbed on hcp/fcc sites can react with gas-phase CO via disproportionation, forming CO_2_ and leaving a C atom behind on the Ir surface.

## Supporting Information Available

Evolution of LEED patterns of CO overlayer structures on Ir(111); PPP and SSP spectra of CO on “defect-rich” Ir(111); fitted results of pressure- and temperature-dependent PPP and SSP spectra of CO on “defect-rich” Ir(111); and fitted results of PPP spectra of CO on “perfect” and “defect-rich” Ir(111) (PDF)
