# Supplementary material for: CO Adsorption and Disproportionation on Smooth and Defect-Rich Ir(111)
Source: J Phys Chem C Nanomater Interfaces. 2022 Apr 8;126(15):6578–89. doi: 10.1021/acs.jpcc.2c01141 (PMC9036526; doi:10.1021/acs.jpcc.2c01141)
Supplement: Supplementary file 1 — jp2c01141_si_001.pdf [file jp2c01141_si_001.pdf]

Supporting Information

CO Adsorption and Disproportionation on Smooth and Defect-Rich Ir(111)

Xia Li[1], Thomas Haunold[1], Stefan Werkovits[1], Laurence D. Marks[2], Peter Blaha[1], Günther Rupprechter[1,*]

[1]Institute of Materials Chemistry, Technische Universität Wien, 1060 Vienna, Austria

[2]Department of Materials Science and Engineering, Northwestern University, Evanston IL 60208, USA

* Corresponding author: guenther.rupprechter@tuwien.ac.at   Phone: +43 (1) 58801-165100

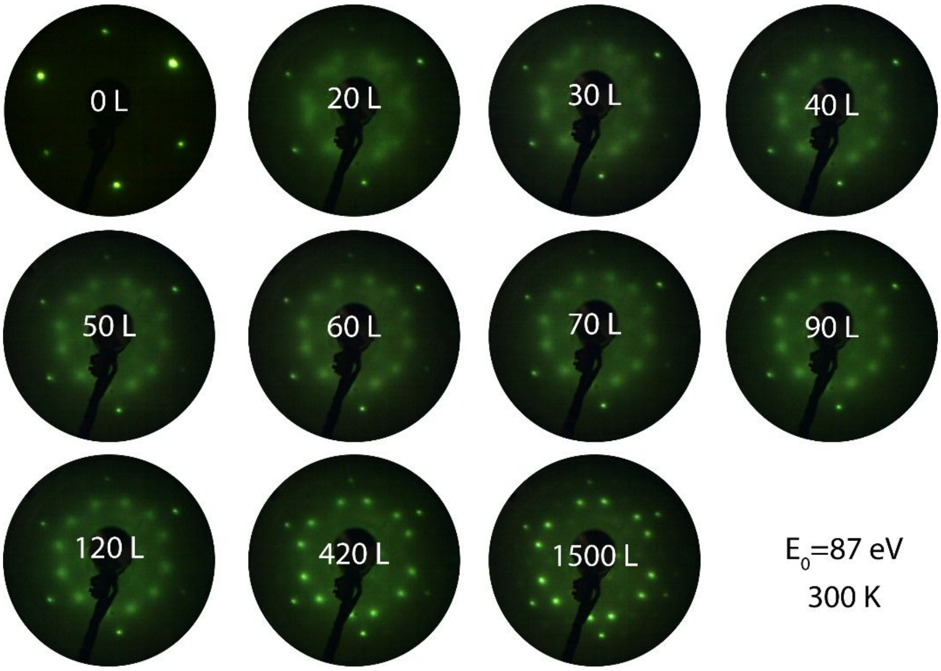

Figure S1 Evolution of LEED patterns at E_0=87 eV of CO overlayer structures on Ir(111) as a function of CO exposure at 300 K. 5 to 120 L (1 Langmuir ≈ 10^{-6} mbar·s) were obtained by dosing CO at 5x10^{-8} mbar, and 420 and 1500 L were achieved by dosing CO at 5x10^{-7} mbar, respectively.

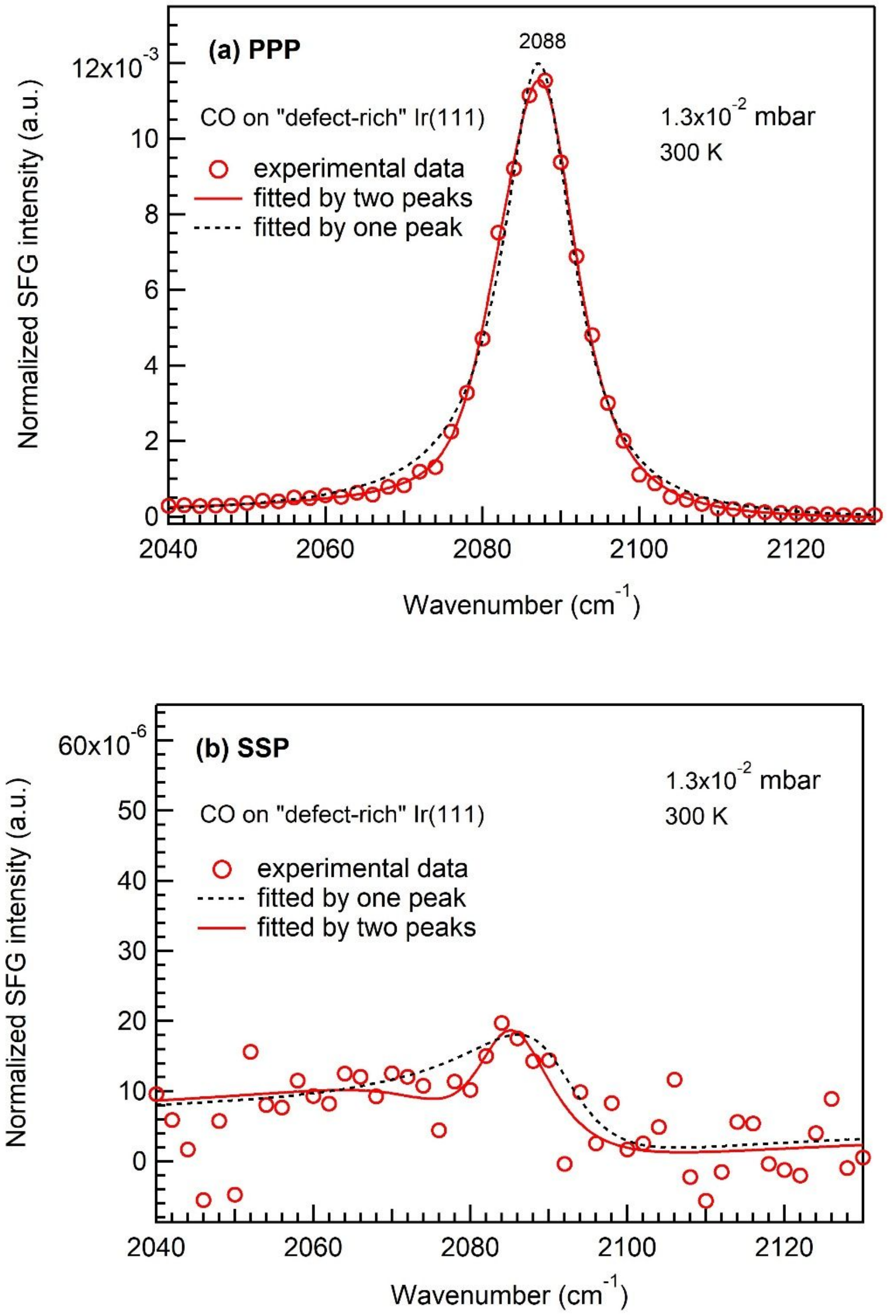

Figure S2 (a) PPP and (b) SSP spectra of CO on "defect-rich" Ir(111) at 1.3x10^{-2} mbar at 300 K fitted by one (~2088 cm^{-1}) and two peaks (~2088 and ~2070 cm^{-1}).

Table S1 Fitted results of pressure- **(Figure 3)** and temperature- (**Figure 4**) dependent PPP and SSP spectra of CO on"defect-rich" Ir(111): peak position, I_{PPP}, I_{SSP}, I_{PPP}/I_{SSP} and tilt angle (θ) as a function of coverage.

P (mbar)	T (K)	$\omega_{IR,1}$ (cm^{-1})	$\omega_{IR,2}$ (cm^{-1})	coverage (ML)	I_{PPP} x10^3 (a.u.)	I_{SSP} x10^5 (a.u.)	I_{PPP}/I_{SSP}	θ (°) (R=0.08)	θ (°) (R=0.07)	θ (°) (R=0.06)
$1.0x10^{-7}$	300	2066	2079	0.56	12.8	2.46	~520	0	8	11
$1.1x10^{-6}$		2070	2081	0.59	14.4	2.75	~524	-	8	11
$1.0x10^{-3}$		2074	2086	0.66	11.8	1.9	~620	-	4	9
0.13		2074	2088	0.68	11.2	2.02	~555	-	7	10
1.0		2080	2090	0.70	12.4	1.52	~820	-	-	4
1.0	500	2066	2079	0.55	11.3	1.89	~590	-	5.5	9.5
	425	2075	2084	0.62	8.9	1.65	~540	-	7	10
	350	2080	2088	0.67	9.3	2.08	~450	6	10	12.5
	300	2080	2090	0.70	14.9	1.85	~805	-	-	5

Table S2 Fitted results of PPP spectra of CO on "perfect" and "defect-rich" Ir(111) (**Figure 10**): peak position and I_{PPP} as a function of coverage, for heat-up and cool-down.

"perfect" Ir(111), 0.013 mbar CO

	heat-up				cool-down			
T (K)	ω_1 (cm^{-1})	ω_2 (cm^{-1})	coverage (ML)	I_{PPP} x10^3 (a.u.)	ω_1 (cm^{-1})	ω_2 (cm^{-1})	coverage (ML)	I_{PPP} x10^3 (a.u.)
575	-	2043±1	0.08±0.1	0.101	-	2043±1	0.05±0.1	0.101
500	-	2054±1	0.15±0.1	1.21	-	2046±1	0.10±0.1	0.238
425	-	2077±1	0.48±0.1	3.02	-	2054±1	0.15±0.1	0.532
350	-	2086±1	0.65±0.1	1.77	-	2063±1	0.23±0.1	0.569
300	-	2091±1	0.72±0.1	1.38	-	2068±1	0.29±0.1	0.592
**"defect-rich"** Ir(111), 1.0 mbar CO								
600	-	-	0.00	0.00	-	-	0.00	0.00
550	2065±1	2074±1	0.44±0.1	3.75	-	-	-	-
500	2066±1	2079±1	0.55±0.1	11.3	2066±1	2060±1	0.19±0.1	0.545
425	2075±1	2084±1	0.62±0.1	8.9	2075±1	2069±1	0.30±0.1	0.281
350	2080±1	2088±1	0.67±0.1	9.3	2080±1	2075±1	0.45±0.1	0.217
300	2080±1	2090±1	0.70±0.1	14.9	2080±1	2078±1	0.55±0.1	0.176
